# Identification of Major Effect QTLs for Agronomic Traits and CSSLs in Rice from Swarna/*Oryza nivara* Derived Backcross Inbred Lines

**DOI:** 10.3389/fpls.2017.01027

**Published:** 2017-06-22

**Authors:** Malathi Surapaneni, Divya Balakrishnan, Sukumar Mesapogu, Krishnam Raju Addanki, Venkateswara Rao Yadavalli, V. G. N. Tripura Venkata, Sarla Neelamraju

**Affiliations:** ICAR-National Professor Project, Indian Institute of Rice ResearchHyderabad, India

**Keywords:** BILs, CSSLs, *O. nivara*, QTL mapping, SSRs, wild species, yield

## Abstract

Backcross inbred lines (BILs) derived from elite x wild crosses are very useful for basic studies and breeding. The aim of this study was to map quantitative trait loci (QTLs) associated with yield and related traits and to identify chromosomal segment substitution lines (CSSLs) from unselected BC_2_F_8_ BILs of Swarna/*Oryza nivara* IRGC81848. In all, 94 BILs were field evaluated in 2 years (wet seasons, 2014 and 2015) for nine traits; days to 50% flowering, days to maturity (DM), plant height (PH), number of tillers, number of productive tillers, panicle weight, yield per plant, bulk yield, and biomass. BILs were genotyped using 111 polymorphic simple sequence repeats distributed across the genome. Fifteen QTLs including 10 novel QTLs were identified using composite interval mapping, Inclusive composite interval mapping and multiple interval mapping (MIM). *O. nivara* alleles were trait-enhancing in 26% of QTLs. Only 3 of 15 QTLs were also reported previously in BC_2_F_2_ of the same cross. These three included the two major effect QTLs for DM and PH detected in both years with 13 and 20% phenotypic variance. Further, a set of 74 CSSLs was identified using CSSL Finder and 22 of these showed significantly higher values than Swarna for five yield traits. CSSLs, 220S for panicle weight and 10-2S with consistent high yield in both years are worthy of large scale field evaluation. The major QTLs and 22 significantly different CSSLs are a useful resource for rice improvement and dissecting yield related traits.

## Introduction

Rice (*Oryza sativa* L.) is the most important cereal food crop cultivated worldwide. The rapid growth in the world’s population, which is expected to reach 9.1 billion by 2050 demands global rice production to be doubled (Ray et al., 2013). Over the years, intensive breeding to develop high yielding varieties quickly using few elite parents has resulted in loss of genetic variability in the cultivars. On the other hand, land races and wild species of rice are sources of abundant genetic variation that can be tapped to increase rice yield sustainably. The wild species of *Oryza* are an important source of genetic variability for tolerance to biotic and abiotic stresses and for improvement of yield as well (Brar and Khush, 1997; Brar and Singh, 2011; Singh K. et al., 2016).

Tanksley and Nelson (1996) proposed advanced backcross quantitative trait loci (AB-QTL) strategy to simultaneously identify and transfer valuable alleles from unadapted germplasm into the elite cultivars and it has been followed in several crops. The annual wild species, *O. nivara* adapted to seasonally dry habitats is the closest progenitor of *O. sativa* (Sharma and Shastry, 1965). It is also a potential source of favorable alleles for agriculturally important traits. One *O. nivara* accession IRGC101508 from Uttar Pradesh, India was identified as the only accession resistant to grassy stunt virus after screening 5000 accessions and 1000 breeding lines (Khush, 1977). *O. nivara* also contributed resistance to bacterial blight (Cheema et al., 2008) and blast (Eizenga et al., 2013). Li et al. (2006) mapped QTLs for domestication traits in F_2_ population derived from CL16/*O. nivara* IRGC80470. QTLs for seedling vigor, yield and quality traits were reported from an advanced backcross population derived from M-202/*O. nivara* IRGC100195 (Eizenga et al., 2015). More recently, a set of 131 ILs were developed from a cross between 93-11 and *O. nivara* W2014 and 65 QTLs were identified for 13 agronomic traits using whole genome resequencing (Ma et al., 2016).

At Indian Institute of Rice Research, two accessions of *O. nivara* IRGC81832 and IRGC81848 which were genetically distinct from 22 other accessions were extensively used for QTL mapping of yield in BC_2_F_2_ and quality traits in BC_2_F_3_ seed (Sarla et al., 2003). Kaladhar et al. (2008) identified 17 major effect QTLs for different yield traits in BC_2_F_2_ population derived from Swarna/*O. nivara* IRGC81832 including *yldp8.1* with LOD score of 8.76 which increased yield by 5.8 g per plant and grain number by 426 grains per plant. Significant yield enhancing QTLs *qyldp2.1, qyldp3.1, qyldp8.1*, *qyldp9.1*, *qyldp11.1* were reported from the other BC_2_F_2_ population derived from Swarna/*O. nivara* IRGC81848 (Swamy, 2009; Swamy et al., 2014). QTLs for stem diameter *qSD7.2*, *qSD8.1*, *qSD9.1*, rachis diameter *qRD9.1* and number of secondary branches *qNSB1.1* were identified as good targets for use in MAS (Swamy et al., 2011). QTLs *qmp1.2, qkw3.1, qkw6.1*, *qklac12.1* for grain quality traits such as milling percentage, kernel width and kernel length after cooking were also identified in these two populations (Swamy et al., 2012). One IL IET21542 (RPBio4918-248) derived from Swarna/*O. nivara* IRGC81848 gave mean yield of 5.5 t/ha for three consecutive years across several locations and a maximum yield of 10.6 t/ha in Coimbatore in wet season 2011 (Annual Progress Report, 2012–2013). It was released as DRR Dhan 40 for three important rice growing states of India – West Bengal, Maharashtra and Tamil Nadu in 2013 (Sarla, 2014). This variety has two QTLs *yld9.1* for yield and *nfg9.1* for number of filled grains from *O. nivara*. Also, six ILs 212S, 215S, 221S, 224S, 228S, and 230S from the same cross were BPH resistant (Lakshmi et al., 2010) and 228S in particular was resistant to multiple pests when evaluated in 10 green house conditions and 46 field tests against 11 pests in multiple resistance screening trials (MRST) (Annual Progress Report, 2012–2013). Two ILs 166S and 75S were tolerant to drought and salinity (Rai et al., 2010). Selected ILs from these two populations were analyzed for G × E interaction and two stable ILs 166S and 14S were identified (Divya et al., 2016). The usefulness of *O. nivara* in improving varieties for yield and other traits is thus quite evident.

Chromosome segment substitution lines (CSSLs), which carry a single or few chromosome segments from the donor in the genetic background of the recurrent parent with the whole donor genome, are ideal for QTL mapping/cloning and also as a genetic resource for pyramiding target segments and breeding (Ali et al., 2010). Several CSSLs have been developed in rice and characterized for yield related traits (Ando et al., 2008; Ookawa et al., 2016). Most of the CSSLs used in these reports were derived from crosses within *O. sativa*. The other cultivated species of rice *O. glaberrima* can also be used to improve *O. sativa* (Sarla and Swamy, 2005). A set of 34 CSSLs were developed from *O. glaberrima* and 105 QTLs for 10 yield traits related to grain yield, plant stature and maturity were identified (Shim et al., 2010).

Chromosomal segment substitution lines have also been developed using wild rice species. CSSLs using *O. rufipogon* helped identify QTLs for yield and related traits (Qiao et al., 2016). A total of 40 CSSLs were developed from *O. longistaminata* in the background of Taichung 65 and evaluated for yield traits (Ramos et al., 2016). Twenty six CSSLs harboring *O. nivara* genomic segments in the genetic background of Koshihikari were developed and evaluated for agriculturally important traits (Furuta et al., 2016). A set of 131 ILs carrying, a total of 767 chromosomal segments from *O. nivara* (W2014) in the genetic background of 93-11 were recently reported (Ma et al., 2016). The *O*. *nivara* allele conferred positive effects at 37% of yield associated QTLs. A new gene-specific InDel marker LQ30 for a gene LOC_Os03g14850 for improved stigma length was developed using a single segment substitution line SSSL14 (Liu et al., 2015). Thus CSSLs can be easily used for gene discovery. We also wanted to know if the major QTLs detected in BC_2_F_2_ remain major effect when the BC_2_F_2_ are advanced to BC_2_F_8_ or do new QTLs become major QTLs with the change in background from BC_2_F_2_ to BC_2_F_8_ of the same cross. Keeping in view, the importance of ILs as a prebreeding material harboring loci for yield enhancing QTLs/genes and CSSLs as a genetic resource for crop improvement, the present study was aimed to (i) detect major QTLs for yield traits in BC_2_F_8_ population derived from Swarna/*Oryza nivara* (ii) compare them with those identified in BC_2_F_2_ from the same cross and (iii) identify a set of complete chromosomal segment substitution lines from BC_2_F_8_ BILs.

## Materials and Methods

### Plant Material

A set of BC_2_F_5_ BILs, derived from a cross between an elite rainfed lowland cultivar Swarna (*O. sativa*) also known as MTU 7029 as a recurrent parent and a wild accession *O. nivara* IRGC81848 as a donor parent was developed by Swamy (2009) and these BILs were self-pollinated in consecutive generations to obtain BC_2_F_8_ families by single panicle selection (Supplementary Table 1).

### Phenotypic Evaluation

Ninety four BC_2_F_8_ lines along with recurrent parent Swarna were grown in wet seasons (*Kharif*) of 2014 and 2015 at Indian Institute of Rice Research (IIRR), Hyderabad. Experiments were conducted using Randomized Complete Block Design (RCBD) with five replications each. The following traits were evaluated. DFF – number of days from sowing to the time that 50% of the plants showed flowering; DM – duration in days from sowing to the time when more than 80% of the grains on the panicles were fully ripened; PH - length in centimeters from the soil surface to the tip of the highest panicle at the time of harvest; Number of tillers per plant (NT) – number of tillers at the time of harvest; Number of productive tillers per plant (NPT) – number of panicle-bearing tillers at the time of harvest; PW – weight of five panicles per plant; YLDP-weight of the harvested seeds per plant; BY weight of dried and cleaned seeds from 30 plants and Biomass (BM) per plant- weight of well-dried mature harvested plants without panicles. LS means were calculated on data pooled by years using PB tools (Version 1.4^1^) and were used in further statistical analysis and QTL mapping.

### Trait Association

Correlation was calculated based on two sample *t*-test with equal variances. CORREL function of the analysis tool pack was used and then imported in excel to find the correlation coefficient between two variables. Correlation among traits was computed at *P* < 0.05 and *P* < 0.01, respectively.

### Genotyping

Total genomic DNA was isolated from fresh leaf samples of 94 BC_2_F_8_ plants and the parents following CTAB method (Doyle and Doyle, 1987). In all, 324 markers were tested for parental polymorphism. One hundred and twenty four SSRs were polymorphic of which 111 gave clear bands and also segregated in BC_2_F_8_ (Supplementary Table 2). PCR was carried out in thermal cycler (G-STORM, United States) with a final reaction volume of 10 μl containing 15 ng of genomic DNA, 1X assay buffer, 200 μM of dNTPs, 1.5 mM MgCl_2_, 10 pmol of forward and reverse primer and 1 unit of Taq DNA polymerase (Thermo Scientific). PCR cycles were programmed as follows: initial denaturation at 94°C for 5 min followed by 35 cycles of 94°C for 30 s, 55°C for 30 s, 72°C for 1 min and a final extension of 10 min at 72°C. Amplified products were resolved in 3% agarose gel prepared in 0.5 × TBE buffer and electrophoresed at 120 V for 2 h. Gels were stained with ethidium bromide and documented using gel documentation system (Alpha Imager, United States).

### Linkage Mapping and QTL Analysis

Linkage map was constructed based on genotypic data of 94 BILs using 111 polymorphic SSR markers on all chromosomes using MAP function (BC_2_RIL) of QTL IciMapping v4.1^2^ using the Kosambi mapping function (Kosambi, 1944). QTL detection was carried out by Composite Interval Mapping, Inclusive Composite Interval Mapping (ICIM) and Single trait Multiple Interval Mapping (SMIM) method in QGene 4.4.0 software (Joehanes and Nelson, 2008). Analysis was undertaken using automatic parameter setting and controlling marker forward stepwise. The threshold of LOD for declaring the presence of significant QTL for each trait was determined using 1000 permutations and α = 0.05 in QGene 4.4.0 software. At α = 0.05, the LOD threshold values ranged from 2.8 to 8.8.

### Identification of CSSL Set

Chromosomal segment substitution lines were identified using genotypic data of 111 polymorphic loci in 94 BILs in the background of the recurrent parent Swarna using the software CSSL Finder^3^. Statistical analysis was carried out using PB tools (Version 1.4^4^) for the test of significance.

## Results

### Phenotypic Evaluation

The mean values of parents and BILs in each year for nine traits and number of BILs showing significant trait increase over recurrent parent Swarna is given in **Table 1**. The highest range of variation was observed for PH, NT, YLDP, and BY in 2014 and for PH, PW, BY, and BM in 2015. Positive transgressive segregation was observed for seven traits in both years compared with Swarna. In 2 years an average of 34% of BILs exhibited 15% to 70% increase over Swarna for PH, PW, YLDP, BY, and BM. Significant pair-wise comparison (at *P* = 0.05) of replicated phenotypic data of the BILs with Swarna as control revealed that BILs 166-9S, 235S, 10-2S, 10-3-4S and 84S had significantly higher yield with mean of 14.43 to 26.40 g per plant but only in 1 year. Likewise, three BILs for DFF and PH, 9 BILs for BM and 30 BILs for PW were significantly different from Swarna but only in 1 year. The highest number of lines (11 lines) with significant improvement over Swarna was observed for the trait PW. One BIL, 220S showed significantly higher PW over Swarna in both years. Two BILs 14-3S and 166-32S had significantly lower PW compared to Swarna in 2014 (data not shown). All traits followed a normal distribution except NT in 2014 and PW in 2015 as shown in Supplementary Figures 1, 2.

**Table 1:** Details of phenotypic traits of parents and mean range in 94 BILs in 2014 and 2015.

S. No	Trait	Swarna	*Oryza nivara* IRGC81848	Range in BILs		Number of BILs showing >15% increase over Swarna	
				2014	2015	2014	2015
(1)	Days to 50% flowering	127	132	91–145	96–126	0	0
(2)	Days to maturity	155	165	121–154	125–154	0	0
(3)	Plant height (cm)	86	123	70– 170	70–162	44	52
(4)	Number of tillers	16	60	2–44	6–25	10	19
(5)	Number of productive tillers	15	51	2–36	4–24	9	14
(6)	Panicle weight (g)	3.1	2.0	1–6	1–5	16	34
(7)	Yield per plant (g)	17	–	2–81	5–32	30	28
(8)	Bulk yield (g)	288	–	42–634	36–400	36	37
(9)	Biomass (g)	25	–	8–86	10–52	37	43

*S. No, serial number; ‘–’, denotes not available*.

### Trait Correlations

Significant correlations were observed among the traits in both the years at *P* < 0.05 and *P* < 0.01 (Supplementary Table 3). In both years, significant positive correlation was observed between DFF and DM; PH with PW, YLDP and BM; NT and NPT; PW and YLDP; YLDP with BY and BM; and BM and BY. Significant negative correlation was observed between PH and NPT in both years. In 2014, DFF was positively correlated with NT and YLDP, but negatively with BY in 2015. NT and NPT showed significant positive correlation with YLDP, BY, and BM only in 2014. In 2015, positive significant correlation was observed between PH and BY; PW and BY, but were negatively correlated in 2014. Negative correlation was observed for DM with PW and BY; PH with NT in both years, but significant only in 2015.

Correlation analysis of both years mean data showed that YLDP has significant positive correlation with PH, NT, NPT, PW, BM, and BY. Significantly correlated traits DFF and DM were negatively correlation with both PW and BY. PH had significant positive correlation with PW, YLDP, and BM but negative correlation with NT and NPT. BM also showed significant positive correlation with PH, NT, NPT, and PW.

### Molecular Characterization of BILs

Considering all the 111 loci and 94 BILs, a total of 7488 alleles were detected covering all chromosomes. Swarna homozygous alleles accounted for 84%, *O. nivara* homozygous alleles 11% and heterozygous alleles 4%. The highest number of *O. nivara* alleles were detected on chromosome 1 (128 alleles), chromosome 2 (120 alleles) and chromosome 8 (108 alleles) and lowest number of *O. nivara* alleles were detected on chromosome 10 (10 alleles). The *O. nivara* introgressions among BILs ranged from 1.15 to 27.0%, with a mean of 10.1%. Number of heterozygotes at any locus ranged from 0 to 40 (42%). RM125 on chromosome 7 followed by RM519 on chromosome 12 showed the highest number of heterozygotes. BIL 138S had was highest number of 24 heterozygotic loci (21% of all loci).

### Construction of Linkage Map and QTL Mapping

**FIGURE 1:**
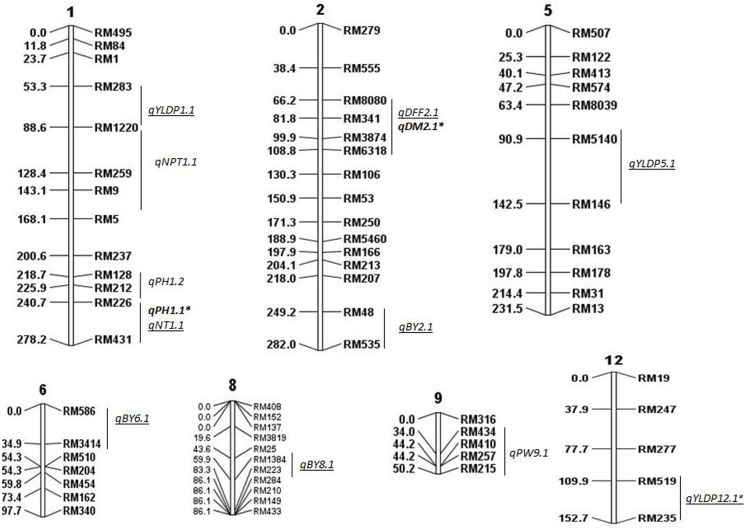
Molecular linkage map of 111 SSRs with position of QTLs for agronomic traits using QGene. Linkage map constructed using ICIM was 1178cM long. DEF, days to 50% flowering; DM, days to maturity; PH, plant height; NT, number of tillers; NPT, number of productive tillers; PW, panicle weight; YLDP, yield per plant; BY, bulk yield; BM, biomass. ‘^∗^’ denotes common QTLs detected in BC_2_F_2_ and BC_2_F_8_. QTLs identified in both years are shown in bold, QTLs identified only in 2015 are underlined.

Linkage map was constructed based on genotypic data of 94 BILs using 111 polymorphic SSR markers on all chromosomes. In all, 15 QTLs were identified for all traits except for BM, considering data of both years using CIM, ICIM, and MIM (**Figure 1**). Five QTLs were identified in 2014 and 10 QTLs in 2015 (**Tables 2**, **3**). Two QTLs were common in both years for DM and PH considering CIM, ICIM and MIM. PV explained by these 15 QTLs ranged from 12 to 56%. *O. nivara* alleles were trait enhancing in 26% of QTLs for 3 traits.

**Table 2:** Trait wise QTLs for agronomic traits detected using 111 SSRs in the BC_2_F_8_ population of Swarna/*O. nivara* in 2014.

S. No	QTL	Chr.	Marker Interval	Peak marker	Method	LOD score	*R*^2^	Additive effect
(1)	***qDM2.1^∗^***	2	RM8080-RM6318	RM3874	ICIM	2.9	13.3	2.7
(2)	***qPH1.1^∗^***	1	RM226-RM431	RM431	CIM	6.4	27.0	-16.3
				RM431	ICIM	3.9	17.5	-11.4
(3)	*qPH1.2*	1	RM128-RM226	RM128	CIM	2.9	13.0	-10.2
(4)	*qNPT1.1*	1	RM1220-RM5	RM259	CIM	3.0	13.0	-2.9
(5)	*qPW9.1*	9	RM434-RM215	RM257	CIM	3.5	13.0	-4.4

*S. No, serial number; Chr., chromosome; DFF, days to 50% flowering; DM, days to maturity; PH, plant height; NT, number of tillers, NPT, number of productive tillers; PW, panicle weight; YLDP, yield per plant; BY, bulk yield; BM, biomass. QTLs in bold are common in both years. ‘^∗^’denotes common QTL in BC_*2*_F_*2*_ and BC_*2*_F_*8*_. LOD, logarithm of odds, positive value indicates additive effect is from Swarna and negative additive value indicates additive effect is from *O. nivara* IRGC81848*.

**Table 3:** Trait wise QTLs for agronomic traits detected using 111 SSRs in the BC_2_F_8_ population of Swarna/*O. nivara* in 2015.

S. No	QTLs	Chr.	Marker Interval	Peak Marker	Method	LOD score	*R*^2^	Additive effect
(1)	*qDFF2.1*	2	RM8080-RM6318	RM3874	ICIM	3.0	12.0	4.6
(2)	***qDM2.1^∗^***	2	RM8080-RM6318	RM3874	CIM	2.9	13.3	2.7
				RM3874	ICIM	2.9	13.3	2.6
(3)	***qPH1.1^∗^***	1	RM226-RM431	RM431	CIM	3.9	17.5	-11.4
				RM431	ICIM	3.9	17.5	-11.4
(4)	*qNT1.1*	1	RM226-RM431	RM226	MIM	5.2	22.5	5.3
(5)	*qYLDP1.1*	1	RM283-RM1220	RM283	MIM	9.3	36.5	24.3
(6)	*qYLDP5.1*	5	RM5140-RM146	RM5140	MIM	11.5	43.1	28.2
(7)	*qYLDP12.1^∗^*	12	RM519-RM235	RM519	MIM	16.7	55.8	34.9
(8)	*qBY2.1*	2	RM48-RM535	RM535	MIM	8.2	33.1	97.5
(9)	*qBY6.1*	6	RM586-RM3414	RM3414	MIM	6.5	27.2	91.7
(10)	*qBY8.1*	8	RM1384-RM223	RM223	MIM	7.5	30.6	94.2

*S. No, serial number; Chr., chromosome; DFF, days to 50% flowering; DM, days to maturity; PH, plant height; NT, number of tillers; NPT, number of productive tillers; PW, panicle weight; YLDP, yield per plant; BY, bulk yield; BM, biomass. QTLs in bold are common in both years. ‘^∗^’denotes common QTL in BC_*2*_F_*2*_ and BC_*2*_F_*8*_. LOD, logarithm of odds, positive value indicates additive effect is from Swarna and negative additive value indicates additive effect is from *O. nivara* IRGC81848*.

### Common QTLs Identified in 2014 and 2015

In all, 15 QTLs were identified, of which only 2 QTLs were common in both years and their PV ranged from 13 to 27%. These were *qDM2.1* and *qPH1.1*. The common QTL *qDM2.1* was identified at chromosomal region RM8080-RM6318 and explained PV of 13% with LOD score 2.9. A major QTL *qPH1.1* at marker interval RM226-RM431 was identified in both years with average LOD of 4.5 explaining PV of 20%. *O. nivara* allele was trait-enhancing in *qPH1.1* and Swarna allele at *qDM2.1* in both years.

### QTLs Identified in 2014

A total of 5 QTLs were identified, of which 2 were also identified in 2015. One QTL *qPH1.2* was identified at RM128-RM226 region with PV of 13%. One QTL each for NPT (*qNPT1.1*) and PW (*qPW9.1*) were identified with PV 13% each and LOD score of 3 and 3.5, respectively. The increasing effect of these three QTLs *qPH1.2, qNPT1.1* and *qPW9.1* was from *O. nivara*.

### QTLs Identified in 2015

In all, 10 QTLs were identified, of which 2 were also identified in 2014. The PV explained by these 10 QTLs ranged from 12 to 56%. Three QTLs each were identified for YLDP and BY. Three QTLs *qYLDP1.1*, *qYLDP5.1*, and *qYLDP12.1* showed PV ranging from 36 to 56% and trait enhancing alleles from Swarna. Of these three QTLs, one major effect QTL *qYLDP12.1* was identified at chromosomal region RM519-RM235 with highest LOD of 16.7 and 56% PV. Three major QTLs *qBY2.1, qBY6.1, and qBY8.1* were identified with PV ranging from 27 to 33%. One QTL each for DFF (*qDFF2.1*) and NT (*qNT1.1*) were identified with PV 12 and 22.5%. Two common QTLs *qDM2.1* and *qPH1.1* were identified in both years.

### Identification of CSSLs

**FIGURE 2:**
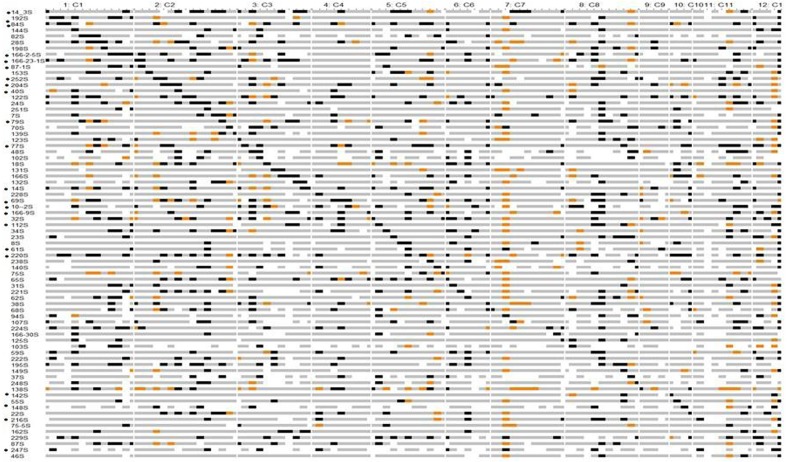
Graphical genotypes of Swarna chromosomal segment substitution lines with *Oryza nivara* segments using 111 SSRs in BC_2_F_8_. Gray – Swarna homozygous, black – *O. nivara* homozygous, orange -heterozygous, white - missing data. ‘^∗^’ indicates CSSL value significantly more than in Swarna for different traits. Details given in Supplementary Table 4.

Chromosomal segment substitution line Finder output showed that 74 CSSLs had homozygous chromosome segments from *O. nivara* substituting Swarna segments (**Figure 2**). These 74 CSSLs together showed 89% coverage of the wild genome based on the 111 SSRs marker data. Small regions on chromosomes 4, 6, 7, 9, 11, and 12 were not represented in the 74 CSSLs. The average number of substituted segments per chromosome in these was 6.5. The number of substituted segments in each CSSL ranged from 2 to 18, with an average of 9.5% (Supplementary Figure 3). Chromosome 1 was represented by 8 CSSLs, chromosome 2 by 13 CSSLs, chromosome 3 by 9 CSSLs and chromosome 10 by 4 CSSLs.

Chromosomal segment substitution line 142S had only three substituted segments and they were on chromosome 10 and showed significantly higher BM than Swarna. Two lines 220S and 166-23-1S showed significantly higher PW than Swarna in both the years.

## Discussion

In the present study, transgressive segregants were obtained with about 15% improvement over Swarna for many yield related traits which indicates that alleles from *O. nivara* were favorable in the genetic background of Swarna in BC_2_F_8_ generation also. It is significant that 10 new QTLs were identified in BC_2_F_8_ which were not detected previously in BC_2_F_2_ population (Swamy et al., 2011, 2014). Of 15 QTLs identified in the study, three QTLs for PH, DM, and YLDP on chromosomes 1, 2 and 12, respectively, were identified in BC_2_F_2_ also.

Most of the QTL regions identified in our study were associated with two or more traits. For example, chromosomal region RM226-RM431 on chromosome 1 had QTLs for PH and NT. Likewise, the chromosomal region RM1220 on chromosome 1 had QTLs for NPT and YLDP; RM8080-RM6318 on chromosome 2 had QTLs for DFF and DM. Correspondingly, there was also significant correlation between DFF and DM in both years; NPT and YLDP in 2015. This may be due to the pleiotropic effects of same QTL alleles/genes controlling PH, NPT and YLDP or presence of two or more adjacent QTL alleles at same locus each controlling a different trait. These QTLs can be used for marker assisted improvement of different traits simultaneously because of their strong and consistent linkage with yield. However, it may be noted that though QTLs for correlated traits were colocalised but the effect of QTLs depends on the genetic context and the direction of their association (Dufey et al., 2015).

### Common QTLs in BC_2_F_2_ and BC_2_F_8_

Yield enhancing QTLs have been reported previously from *O. nivara* in BC_2_F_2_ families (Kaladhar et al., 2008; Swamy et al., 2011, 2014). A total of 28 QTLs for yield traits were reported in BC_2_F_2_ population derived from the same cross of Swarna/*O. nivara* IRGC81848 and 78% of the loci from *O. nivara* were trait enhancing (Swamy et al., 2014). Three common QTLs for traits PH (*qPH1.1*- RM431) on chromosome 1, DM (*qDM2.1*-RM3874) on chromosome 2, and YLDP (*qYLDP12.1*- RM519) on chromosome 12 were identified in both BC_2_F_2_ (previous study) and BC_2_F_8_ (present study) and these three are thus significant major effect QTLs. The first common QTL *qPH1.1* is close to the well known semi- dwarf locus *sd-1*, the green revolution gene and explained PV of 22 and 17.5% in 2014 and 2015, respectively. Thus, it shows the robustness of our study as these major QTLs were identified in BC_2_F_2_ and BC_2_F_8_ and in both years. The major effect QTL *qPH1.1* was identified in both years and in both generations (BC_2_F_8_ and BC_2_F_2_) indicating that it is also a stable QTL. RM431 the peak marker is within the region of QTL *qDTY1.1* reported previously for yield under drought and being transferred into submergence tolerant versions of three high yielding mega rice varieties Swarna-Sub1, Samba Mahsuri-Sub1, and IR 64-Sub1 using MAS (Vikram et al., 2011; Singh R. et al., 2016). In our study, *O. nivara* allele of *qPH1.1* increases PH. The semi dwarfing gene *sd-1*, has been widely utilized in rice production, however, attention has to be paid to breed taller plants with strong culm to enhance plant yield by increasing biomass (Han et al., 2017). They identified QTLs for PH in a RIL population derived from Zhenshan 97 and Xizang 2. Two QTLs *qph1* and *qph7.1* detected in 3 years, explained 13% PV with large additive effect of 12 cm from Xizang 2 allele. In our study, *O. nivara* alleles increased PH by 11 cm in both the years. RM431 flanks PH QTLs reported previously from elite/ wild crosses using accessions of *O. nivara*, *O. rufipogon* and from landraces also (Wickneswari et al., 2012; Eizenga et al., 2013; Mohammadi et al., 2013). In addition to height, RM431 was linked with QTLs for grain number/panicle, harvest index (Li et al., 2012). This indicates that alleles at the vicinity of RM431 are associated with different yield related traits in different genetic backgrounds.

The second common QTL *qDM2.1* at chromosomal region RM3874 falls within a metaQTL *MQTL2.3* reported for PW (Swamy and Sarla, 2011; Swamy et al., 2011). The third common QTL *qYLDP12.1* at RM519 region also harbors QTLs *qrd12.1* for rachis diameter, *qnpt12.1* for number of productive tillers*, qnsp12.1* for number of spikelets and *qnfg12.1* for number of filled grains (Swamy, 2009; Swamy et al., 2014). There are two previous reports on identification of common QTLs in BC_2_F_2_ and later generations. Rangel et al. (2008) identified one common QTL for grain yield on chromosome 1 at RM1 locus from BG90-2/RS-16 (*O. glumaepatula*) in both BC_2_F_2_ and BC_2_F_8_. Similarly, Wickneswari and Bhuiyan (2014) identified two common QTLs *qSPL-1-1* and *qSPL-8* for spikelets per plant in BC_2_F_2_ and BC_2_F_5_ from MR219/*Oryza rufipogon* Griff. IRGC105491.

### QTL Regions in BC_2_F_8_ Which Were Linked with Different Agronomic Traits in BC_2_F_2_

Comparing QTLs in BC_2_F_8_ with BC_2_F_2_ we found there were few other chromosomal regions common in BC_2_F_8_ and BC_2_F_2_ but linked with different traits. In BC_2_F_8,_ RM434 was linked with *qPW9.1* where as in BC_2_F_2_ RM434 was linked with *qyldp9.1*. Likewise, RM128 was linked with *qPH1.2* in BC_2_F_8_ and *qnpt1.1* in BC_2_F_2_; RM223 for *qBY8.1* in BC_2_F_8_ and *qnfg8.1*and *qyldp8.1* in BC_2_F_2_; RM519 for *qYLDP12.1* in BC_2_F_8_ and *qnpt12.1, qnsp12.1* and *qnfg12.1* in BC_2_F_2_. There could be several reasons for this observation. Earlier, 100 SSRs were used to genotype 227 BC_2_F_2_ mapping population whereas in this study 111 different SSRs were used to genotype 94 BC_2_F_8_ BILs and only 37 markers were common with the previous study. The detection of novel QTLs in BC_2_F_8_ might be due to changes in genetic background from BC_2_F_2_ to BC_2_F_8_ or differences in the size of mapping population and the distribution of SSR markers used in the two studies. Also environment conditions were different in the two generations and this might have contributed to differences in phenotype and QTLs.

Considering that the available population size was small, the phenotypic variation explained by QTLs is quite likely to be overestimated. However, since many of the major effect QTLs detected in our study were also identified for the same or different trait previously, it indicates the robustness and importance of these QTLs in regulating the phenotype of yield associated traits. The significant QTLs identified in this study can be further evaluated for use in marker assisted transfer to adapted varieties to improve not only yield but several related traits as QTLs for drought and salinity are being transferred into high yielding mega varieties (Singh R. et al., 2016).

### Identification of CSSLs

Chromosomal segment substitution line are an important genetic resource in rice to discover novel genes by focusing on small chromosomal regions (Ali et al., 2010; Subudhi et al., 2015; Ramos et al., 2016). A set of 74 CSSLs with substituted chromosomal segments of *O. nivara* in the genetic background of Swarna were identified. Graphical genotypes of CSSL library revealed 89% coverage of *O. nivara* genome. A few chromosomal regions were not represented by substituted segments on chromosomes 4, 7, 9, and 11. This could be due to less number of BILs used or the presence of lethal alleles, hybrid sterility and gametophyte lethal genes or even low recombination in these chromosomal locations. Introgression of *O. nivara* chromosomal segments in the genetic background of 93-11 was reported by Ma et al. (2016). The coverage of the *O*. *nivara* genome by the ILs was 94.96%. Several sets of CSSLs have been developed using other wild species such as *O. rufipogon* (Furuta et al., 2014; Qiao et al., 2016; Ogawa et al., 2016), *O. minuta* (Guo et al., 2013), *O. meridionalis* (Arbelaez et al., 2015), *O. longistaminata* (Ramos et al., 2016). A set of 198 CSSLs was developed from a cross between 93-11 and *O. rufipogon*, and introgressed segments covered 84.9% of the wild rice genome (Qiao et al., 2016). Subudhi et al. (2015) developed 74 CSSLs covering 99% of the weedy rice donor PSRR-1 genome. In their study, donor segments per line ranged from 1 to 3 with 64% of CSSLs with single homozygous donor segments. In our study number of donor segments per line ranged from 3 to 18 as CSSLs were identified from a set of available BILs at BC_2_F_8_ generation without MAS. A few backcrosses can be made along with MAS of target regions to recover more background genome of this set or selected CSSLs.

In our previous studies, two stable lines 166S and 14S were reported to be efficient in compartmentalization of Na+ in leaf tissue and grain yield of 166S was least affected by salt stress (Divya et al., 2016; Pushpalatha et al., 2016). Also, three lines 24S, 70S, 14-3S were identified as heat tolerant lines for spikelet fertility and YLDP in both wet and dry seasons (Prasanth et al., 2016). In present study, these five lines 14S, 24S, 70S, 166S and 14-3S were part of the set of CSSLs and they were found to have 11.5–18.6% chromosomal segments from *O. nivara*. Thus, several elite BILs such as these can be used in prebreeding programs for identification of candidate genes for different yield related traits. Among the CSSLs, 220S showed significantly higher PW over the parent Swarna in both years. 10-2S showed highest YLDP in both years over Swarna. These two lines, 220S and 10-2S showed significantly higher yield than Swarna, so they can be used to dissect target QTL regions. In addition, another 20 CSSLs showed different yield traits as significantly higher than Swarna and these marker defined CSSLs can be further utilized for fine mapping.

## Conclusion

Our results provide evidence that *O. nivara* has novel, stable, major effect QTL alleles for PH, DM not only in BC_2_F_2_ but even in BC_2_F_8_. ILs with *O. nivara* alleles for increasing PH or decreasing DM are potential donors for transfer into other popular lines. The set of 74 CSSLs is being phenotyped for other agronomic traits including disease and pest resistance and is an important genetic resource to discover novel alleles for several traits in rice.

## Ethics Statement

The authors declare that the experiments comply with the current laws of the country in which they were performed and in compliance with ethical standards.

## Author Contributions

SN and DB conceived and planned the work. MS, KA, VY, and SM performed phenotypic and genotypic screening. MS, DB, and TV analyzed the data. MS, DB, and SN drafted the manuscript.

## Conflict of Interest Statement

The authors declare that the research was conducted in the absence of any commercial or financial relationships that could be construed as a potential conflict of interest. The reviewer MR and handling Editor declared their shared affiliation, and the handling Editor states that the process met the standards of a fair and objective review.

## Footnotes

1 http://bbi.irri.org/products

2 www.isbreeding.net

3 http://mapdisto.free.fr/CSSLFinder/

4 http://bbi.irri.org/products

## Abbreviations

BILs: backcross inbred lines
BM: biomass
BY: bulk yield
CSSLs: chromosome segment substitution lines
DFF: days to 50% flowering
DM: days to maturity
IL: introgression line
MAS: marker assisted selection
MIM: multiple interval mapping
NT: number of tillers
NPT: number of productive tillers
PH: plant height
PW: panicle weight
QTLs: quantitative trait loci
SSRs: simple sequence repeats
YLDP: yield per plant.

## References

[B1] Ali M. L. Sanchez P. L. Yu S. Lorieux M. Eizenga G. C. (2010). Chromosome segment substitution lines: a powerful tool for the introgression of valuable genes from *Oryza* wild species into cultivated rice (*O. sativa* L.). *Rice.* 3 218–234. 10.1007/s12284-010-9058-3

[B2] Ando T. Yamamoto T. Shimizu T. Ma X. Shomura A. Takeuchi Y. (2008). Genetic dissection and pyramiding of quantitative traits for panicle architecture by using chromosomal segment substitution lines in rice. *Theor. Appl. Genet.* 116 881–890. 10.1007/s00122-008-0722-6 18274726

[B3] Annual Progress Report (2012–2013). *All India Coordinated Rice Improvement Project (AICRIP)*. Hyderabad: Directorate of Rice Research.

[B4] Arbelaez J. D. Moreno L. T. Singh N. Tung C. W. Maron L. G. Ospina Y. (2015). Development and GBS-genotyping of introgression lines (ILs) using two wild species of rice, *O. meridionalis* and *O. rufipogon* in a common recurrent parent, *O. sativa* cv. Curinga. *Mol. Breed.* 35 81 10.1007/s11032-015-0276-7 PMC4328105 25705117

[B5] Brar D. S. Khush G. S. (1997). Alien introgression in rice. *Plant Mol. Biol.* 35 35–47. 10.1023/A:1005825519998 9291958

[B6] Brar D. S. Singh K. (2011). “Oryza,” in *Book: Wild Crop Relatives: Genomic and Breeding Resources: Cereals* Vol. 1 ed. Kole C. (Berlin: Springer Science and Business Media), 321–365.

[B7] Cheema K. K. Grewal N. K. Vikal Y. Sharma R. Lore J. S. Das A. (2008). A novel bacterial blight resistance gene from *Oryza nivara* mapped to 38 kb region on chromosome 4L and transferred to *Oryza sativa* L. *Genet. Res.* 90 397–407. 10.1017/S0016672308009786 19061530

[B8] Divya B. Subrahmanyam D. Badri J. Raju A. K. Rao V. Y. Kavitha B. (2016). Genotype × environment interactions of yield traits in backcross introgression lines derived from *Oryza sativa* cv. Swarna /*Oryza nivara*. *Front. Plant Sci.* 7:1530 10.3389/fpls.2016.01530 PMC5070172 27807437

[B9] Doyle J. J. Doyle J. L. (1987). A rapid DNA isolation procedure for small quantities of fresh leaf tissue. *Phytochem. Bull.* 19 11–15.

[B10] Dufey I. Draye X. Lutts S. Lorieux M. Martinez C. Bertin P. (2015). Novel QTLs in an interspecific backcross *Oryza sativa* x *Oryza glaberrima* for resistance to iron toxicity in rice. *Euphytica* 204 609–625. 10.1007/s10681-014-1342-7

[B11] Eizenga G. C. Neves P. C. F. Bryant R. J. Agrama H. A. Mackill D. J. (2015). Evaluation of a M-202 x *Oryza nivara* advanced backcross mapping population for seedling vigor, yield components and quality. *Euphytica* 208 157–171. 10.1007/s10681-015-1613-y

[B12] Eizenga G. C. Prasad B. Jackson A. K. Jia M. H. (2013). Identification of rice sheath blight and blast quantitative trait loci in two different *O. sativa* x *O. nivara* advanced backcross populations. *Mol. Breed.* 31 889–907. 10.1007/s11032-013-9843-y

[B13] Furuta T. Uehara K. Angeles-Shim R. B. Shim J. Ashikari M. Takashi T. (2014). Development and evaluation of chromosome segment substitution lines (CSSLs) carrying chromosome segments derived from *Oryza rufipogon* in the genetic background of *Oryza sativa* L. *Breed. Sci.* 63 468–475. 10.1270/jsbbs.63.468 24757386 PMC3949583

[B14] Furuta T. Uehara K. Angeles-Shim R. B. Shim J. Nagai K. Ashikari M. (2016). Development of chromosome segment substitution lines (CSSLs) harbouring *Oryza nivara* genomic segments in Koshihikari and evaluation of yield related traits. *Breed. Sci.* 66 845–850. 10.1270/jsbbs.16131 28163601 PMC5282765

[B15] Guo S. Wei Y. Li X. Liu K. Huang F. Chen C. (2013). Development and identification of introgression lines from cross of *Oryza sativa* and *Oryza minuta*. *Rice Sci.* 20 95–102. 10.1016/S1672-6308(13)60111-0

[B16] Han Z. Hu W. tan C. Xing Y. (2017). QTLs for heading date and plant height under multiple environments in rice. *Genetica* 145 67–77. 10.1007/s10709-016-9946-6 28070759

[B17] Joehanes R. Nelson J. C. (2008). QGene 4.0, an extensible Java QTL-analysis platform. *Bioinformatics* 23 2788–2789. 10.1093/bioinformatics/btn523 18940826

[B18] Kaladhar K. Swamy B. P. M. Babu A. P. Reddy C. S. Sarla N. (2008). Mapping quantitative trait loci for yield traits in BC2F2 population derived from Swarna x O. *nivara* cross. *Rice Genet. Newsl.* 24 34–36. 10.1093/jhered/esr145

[B19] Khush G. S. (1977). Disease and insect resistance in rice. *Adv. Agron.* 29 265–341. 10.1016/S0065-2113(08)60221-7

[B20] Kosambi D. D. (1944). The estimation of map distance from recombination values. *Ann. Eugen.* 12 172–175. 10.1111/j.1469-1809.1943.tb02321.x

[B21] Lakshmi J. V. Swamy B. P. M. Kaladhar K. Sarla N. (2010). BPH resistance in introgression lines of Swarna / O. *nivara* and KMR3 / *O. rufipogon*. *DRR News Lett.* 8 4.

[B22] Li C. Zhou A. Sang T. (2006). Genetic analysis of rice domestication syndrome with the wild annual species, *Oryza nivara. New Phytol.* 170 185–194. 10.1111/j.1469-8137.2005.01647.x 16539615

[B23] Li X. Yan W. Agrama H. Jia L. Jackson A. Moldenhauer K. (2012). Unraveling the complex trait of harvest index with association mapping in rice (*Oryza sativa* L.). *PLoS ONE* 7:e29350 10.1371/journal.pone.0029350 PMC3264563 22291889

[B24] Liu Q. Qin J. Li T. Liu E. Fan D. Edzesi W. M. (2015). Fine mapping and candidate gene analysis of *qSTL3*, a stigma length-conditioning locus in rice (*Oryza sativa* L.). *PLoS ONE* 10:e0127938 10.1371/journal.pone.0127938 PMC4452489 26030903

[B25] Ma X. Fu Y. Zhao X. Jiang L. Zhu Z. Gu P. (2016). Genomic structure analysis of a set of *Oryza nivara* introgression lines and identification of yield associated QTLs using whole genome resequencing. *Sci. Rep.* 6:27425 10.1038/srep27425 PMC4890301 27251022

[B26] Mohammadi R. Mendioro M. S. Diaz G. Q. Gregorio G. B. Singh R. K. (2013). Mapping quantitative trait loci associated with yield and yield components under reproductive stage salinity stress in rice (*Oryza sativa* L.). *J. Gen.* 92 433–443. 10.1007/s12041-013-0285-4 24371165

[B27] Ogawa S. Valencia M. O. Lorieux M. Arbelaez J. D. McCouch S. Ishitani M. (2016). Identification of QTLs associated with agronomic performance under nitrogen-deficient conditions using chromosome segment substitution lines of a wild rice relative, *Oryza rufipogon. Acta Physiol. Plant.* 38 103 10.1007/s11738-016-2119-5

[B28] Ookawa T. Aoba R. Yamamoto T. Ueda T. Takai T. Fukuoka S. (2016). Precise estimation of genomic regions controlling lodging resistance using a set of reciprocal chromosome segment substitution lines in rice. *Sci. Rep.* 28:30572 10.1038/srep30572 PMC4964586 27465821

[B29] Prasanth V. V. Basava K. R. Babu M. S. Venkata T. V. G. N. Rama Devi S. J. S. Mangrauthia S. K. (2016). Field level evaluation of rice introgression lines for heat tolerance and validation of markers linked to spikelet fertility. *Physiol. Mol. Biol. Plants* 22 179 10.1007/s12298-016-0350-6 PMC4938818 27436910

[B30] Pushpalatha G. Ajay J. Parmar B. Rao A. R. Sreenu K. Mishra P. (2016). Identification of salt tolerant rice lines among interspecific BILs developed by crossing *Oryza sativa* /*O. rufipogon* and *O. sativa* /*O. nivara*. *Aust. J. Crop. Sci.* 10 220–228.

[B31] Qiao W. Qi L. Cheng Z. Su L. Li J. Sun Y. (2016). Development and characterization of chromosome segment substitution lines derived from *Oryza rufipogon* in the genetic background of *O. sativa* spp. *indica* cultivar 93-11. *BMC Genomics* 17:580 10.1186/s12864-016-2987-5 PMC4979106 27507407

[B32] Rai V. Sreenu K. Puspalatha B. Prasad Babu A. Brajendra P. Sandhya G. (2010). Swarna / *O. nivara* and KMR3 / *O. rufipogon* introgression lines tolerant to drought and salinity. *DRR NewsLetter* 8 4.

[B33] Ramos J. M. Furuta T. Uehara K. Chihiro N. Angeles-Shim R. B. Shim J. (2016). Development of chromosome segment substitution lines (CSSLs) of *O. longistaminata* A. Chev. & Rohr in the background of the elite japonica rice cultivar, Taichung 65 and their evaluation for yield traits. *Euphytica* 210 151–163. 10.1007/s10681-016-1685-3

[B34] Rangel P. N. Brondani R. P. V. Rangel P. H. N. Brondani C. (2008). Agronomic and molecular characterization of introgression lines from the interspecific cross *Oryza sativa* (BG90-2) x *Oryza glumaepatula* (RS-16). *Genet. Mol. Res.* 7 184–195. 10.4238/vol7-1gmr406 18393222

[B35] Ray D. K. Mueller N. D. West P. C. Foley J. A. (2013). Yield trends are insufficient to double global crop production by 2050. *PLoS ONE* 8:e66428 10.1371/journal.pone.0066428 PMC3686737 23840465

[B36] Sarla N. (2014). DRR Dhan40-with yield enhancing QTLs from wild species. *DRR Newsletter* 12 2.

[B37] Sarla N. Bobba S. Siddiq E. A. (2003). ISSR and SSR markers based on AG and GA repeats delineate geographically diverse *Oryza nivara* accessions and reveal rare alleles. *Curr. Sci.* 84 683–690.

[B38] Sarla N. Swamy B. P. M. (2005). *Oryza glaberrima*: a source for the improvement of *Oryza sativa*. *Curr. Sci.* 89 955–963.

[B39] Sharma S. D. Shastry S. V. S. (1965). Taxonomic studies in genus *Oryza* L. III. *O. rufipogon* Griff. sensu stricto and *O. nivara* Sharma et Shastry nom. nov. *Ind. J. Genet. Plant Breed.* 25 157–167.

[B40] Shim R. A. Angeles E. R. Ashikari M. Takashi T. (2010). Development and evaluation of *Oryza glaberrima* Steud. chromosome segment substitution lines (CSSLs) in the background of *O. sativa* L.Cv. Koshihikari. *Breed. Sci.* 60 613–619. 10.1270/jsbbs.60.613

[B41] Singh K. Neelam K. Kaur A. Kaur K. (2016). “Rice,” in *Broadening the Genetic Base of Grain Cereals*, eds Sing M. Kumar S. (New Delhi: Springer India), 27–65. 10.1007/978-81-322-3613-9_3

[B42] Singh R. Singh Y. Xalaxo S. Verulkar S. Yadav N. Singh S. (2016). From QTL to variety-harnessing the benefits of QTLs for drought, flood and salt tolerance in mega rice varieties of India through a multi-institutional network. *Plant Sci.* 242 278–287. 10.1016/j.plantsci.2015.08.008 26566845

[B43] Subudhi P. K. De Leon T. Singh P. K. Parco A. Cohn M. A. Sasaki T. (2015). A chromosome segment substitution library of weedy rice for genetic dissection of complex agronomic and domestication traits. *PLoS ONE* 10:e0130650 10.1371/journal.pone.0130650 PMC4472838 26086245

[B44] Swamy B. P. M. (2009). *Genome wide Mapping of Quantitative Trait Loci (QTLs) for Yield and Grain Quality Traits in O. sativa cv Swarna x O. nivara Backcross Population*. Ph.D Thesis, Osmania University, Hyderabad, India.

[B45] Swamy B. P. M. Kaladhar K. Ramesha M. S. Viraktamath B. C. Sarla N. (2011). Molecular mapping of QTLs for yield and related traits in *Oryza sativa* cv Swarna x *O. nivara* (IRGC81848) backcross population. *Rice Sci.* 18 178–186. 10.1016/S1672-6308(11)60025-5

[B46] Swamy B. P. M. Kaladhar K. Reddy A. G. Viraktamath B. C. Sarla N. (2014). Mapping and introgression of QTL for yield and related traits in two backcross populations derived from *Oryza sativa* cv. Swarna and two accessions of *O. nivara*. *J. Genet.* 93 643–653. 10.1007/s12041-014-0420-x 25572223

[B47] Swamy B. P. M. Kaladhar K. Shobha Rani N. Prasad G. S. V. Viraktamath B. C. Reddy A. G. (2012). QTL analysis for grain quality traits in two BC2F2 populations derived from crosses between *Oryza sativa* cv. Swarna and two accessions of *O. nivara*. *J. Hered.* 103 442–452. 10.1093/jhered/esr145 22312119

[B48] Swamy B. P. M. Sarla N. (2011). Meta-analysis of yield QTLs derived from inter-specific crosses of rice reveals consensus regions and candidate genes. *Plant Mol. Biol. Rep.* 29 663–680.

[B49] Tanksley S. D. Nelson J. C. (1996). Advanced backcross QTL analysis; a method for simultaneous discovery and transfer of valuable QTL from unadapted germplasm into elite breeding lines. *Theor. Appl. Genet.* 92 191–203. 10.1007/BF00223376 24166168

[B50] Vikram P. Swamy B. P. M. Dixit S. Ahmed H. U. Sta Cruz M. T. Singh A. K. (2011). *qDTY1*.1, a major QTL for rice grain yield under reproductive-stage drought stress with a consistent effect in multiple elite genetic backgrounds. *BMC Genet.* 12:89 10.1186/1471-2156-12-89 PMC3234187 22008150

[B51] Wickneswari R. Bhuiyan M. A. R. (2014). Exploiting wild accessions for development of high yielding new rice genotypes. *Malays. Appl. Biol.* 43 89–95.

[B52] Wickneswari R. Bhuiyan M. A. R. Kalluvettankuzhy K. S. Lim L. S. Thomson M. J. Narimah M. K. (2012). Identification and validation of quantitative trait loci for agronomic traits in advanced backcross breeding lines derived from *Oryza rufipogon* x *Oryza sativa* cultivar MR219. *Plant Mol. Biol. Rep.* 30 929–939. 10.1007/s11105-011-0404-4

