# Supplementary material for: Identification of Major Effect QTLs for Agronomic Traits and CSSLs in Rice from Swarna/Oryza nivara Derived Backcross Inbred Lines
Source: Front Plant Sci. 2017 Jun 22;8:1027. doi: 10.3389/fpls.2017.01027 (PMC5480306; doi:10.3389/fpls.2017.01027)
Supplement: Supplementary file 1 [file Table_1.DOCX]

**Identification of major effect QTLs for agronomic traits and CSSLs in rice from Swarna/*Oryza nivara* derived backcross inbred lines**



**Supplementary Table** **1**. List of 94 BC_2_F_8_ BILs derived from Swarna / *O. nivara* used in the study

| **S. No** | **BIL** | **S.No** | **BIL** | **S.No** | **BIL** |
| --- | --- | --- | --- | --- | --- |
| 1 | 7S | 33 | 79S | 65 | 204S |
| 2 | 8S | 34 | 82S | 66 | 212S |
| 3 | 14S | 35 | 84S | 67 | 216S |
| 4 | 15S | 36 | 87S | 68 | 218S |
| 5 | 16S | 37 | 94S | 69 | 220S |
| 6 | 18S | 38 | 102S | 70 | 221S |
| 7 | 22S | 39 | 103S | 71 | 222S |
| 8 | 23S | 40 | 107S | 72 | 224S |
| 9 | 24S | 41 | 112S | 73 | 227S |
| 10 | 28S | 42 | 122S | 74 | 228S |
| 11 | 31S | 43 | 123S | 75 | 229S |
| 12 | 32S | 44 | 125S | 76 | 230S |
| 13 | 34S | 45 | 129S | 77 | 231S |
| 14 | 37S | 46 | 131S | 78 | 233S |
| 15 | 38S | 47 | 132S | 79 | 235S |
| 16 | 40S | 48 | 136S | 80 | 238S |
| 17 | 45S | 49 | 138S | 81 | 244S |
| 18 | 46S | 50 | 139S | 82 | 247S |
| 19 | 48S | 51 | 140S | 83 | 248S |
| 20 | 50S | 52 | 142S | 84 | 251S |
| 21 | 53S | 53 | 144S | 85 | 252S |
| 22 | 55S | 54 | 148S | 86 | 10-2S |
| 23 | 59S | 55 | 149S | 87 | 10-3-4S |
| 24 | 61S | 56 | 153S | 88 | 14-3S |
| 25 | 62S | 57 | 159S | 89 | 75-5S |
| 26 | 65S | 58 | 162S | 90 | 87-1 S |
| 27 | 68S | 59 | 166S | 91 | 166-2-5S |
| 28 | 69S | 60 | 190S | 92 | 166-9S |
| 29 | 70S | 61 | 192S | 93 | 166-23-1S |
| 30 | 71S | 62 | 195S | 94 | 166-30S |
| 31 | 75S | 63 | 198S |  |  |
| 32 | 77S | 64 | 202S |  |  |

S. No – Serial Number, BIL- Backcross inbred line
