# Supplementary material for: Identification of Major Effect QTLs for Agronomic Traits and CSSLs in Rice from Swarna/Oryza nivara Derived Backcross Inbred Lines
Source: Front Plant Sci. 2017 Jun 22;8:1027. doi: 10.3389/fpls.2017.01027 (PMC5480306; doi:10.3389/fpls.2017.01027)
Supplement: Supplementary file 2 [file Table_2.DOCX]

**Identification of major effect QTLs for agronomic traits and CSSLs in rice from Swarna/*Oryza nivara* derived backcross inbred lines**



**Supplementary Table 2**. List of polymorphic SSRs used in the study

| S. No | SSR | Chr. | Forward sequence | Reverse sequence |
| --- | --- | --- | --- | --- |
| 1 | RM495 | 1 | AATCCAAGGTGCAGAGATGG | CAACGATGACGAACACAACC |
| 2 | RM84* | 1 | TAAGGGTCCATCCACAAGATG | TTGCAAATGCAGCTAGAGTAC |
| 3 | RM1* | 1 | GCGAAAACACAATGCAAAAA | GCGTTGGTTGGACCTGAC |
| 4 | RM283 | 1 | GTCTACATGTACCCTTGTTGGG | CGGCATGAGAGTCTGTGATG |
| 5 | RM1220 | 1 | TGCTTCCTGCAGGGGTATAG | GGCAATAGCTAGCAAGGCAG |
| 6 | RM259 | 1 | TGGAGTTTGAGAGGAGGG | CTTGTTGCATGGTGCCATGT |
| 7 | RM9* | 1 | GGTGCCATTGTCGTCCTC | ACGGCCCTCATCACCTTC |
| 8 | RM5* | 1 | TGCAACTTCTAGCTGCTCGA | GCATCCGATCTTGATGGG |
| 9 | RM237 | 1 | CAAATCCCGACTGCTGTCC | TGGGAAGAGAGCACTACAGC |
| 10 | RM128 | 1 | TGATTTCTTGGAAGCGAAGAGTGAGG | CCTCCTTGTGCTCAGCCATGC |
| 11 | RM212 | 1 | CCACTTTCAGCTACTACCAG | CACCCATTTGTCTCTCATTATG |
| 12 | RM226 | 1 | GAAGCTAAGGTCTGGGAGAAACC | AATGGCCTTAACCAAGTAGGATGG |
| 13 | RM431* | 1 | TCCTGCGAACTGAAGAGTTG | AGAGCAAAACCCTGGTTCAC |
| 14 | RM279 | 2 | GCGGGAGAGGGATCTCCT | GGCTAGGAGTTAACCTCGCG |
| 15 | RM555 | 2 | TTGGATCAGCCAAAGGAGAC | CAGCATTGTGGCATGGATAC |
| 16 | RM8080 | 2 | CAGTTTCAGTTTCAGGTCAGT | CTGATGCTGATCACCTGA |
| 17 | RM341 | 2 | CAAGAAACCTCAATCCGAGC | CTCCTCCCGATCCCAATC |
| 18 | RM3874* | 2 | TGGGTGATCTTAGTTTGGCC | AATGTGCCTGCACATGTCAC |
| 19 | RM6318 | 2 | TGCTGCTTCTGTCCAGTGAG | GGATCATAACAAGTGCCTCG |
| 20 | RM106* | 2 | CGTCTTCATCATCGTCGCCCCG | GGCCCATCCCGTCGTGGATCTC |
| 21 | RM53 | 2 | ACGTCTCGACGCATCAATGG | CACAAGAACTTCCTCGGTAC |
| 22 | RM250* | 2 | GGTTCAAACCAAGCTGATCA | GATGAAGGCCTTCCACGCAG |
| 23 | RM5460 | 2 | AAGAGAACAAGCCATGGTGC | GCCTTTTCTTGCCTTTGGAC |
| 24 | RM166* | 2 | GGTCCTGGGTCAATAATTGGGTTACC | TTGCTGCATGATCCTAAACCGG |
| 25 | RM213 | 2 | ATCTGTTTGCAGGGGACAAG | AGGTCTAGACGATGTCGTGA |
| 26 | RM207 | 2 | CCATTCGTGAGAAGATCTGA | CACCTCATCCTCGTAACGCC |
| 27 | RM48 | 2 | TGTCCCACTGCTTTCAAGC | CGAGAATGAGGGACAAATAACC |
| 28 | RM535* | 2 | ACTACATACACGGCCCTTGC | CTACGTGGACACCGTCACAC |
| 29 | RM231 | 3 | CCAGATTATTTCCTGAGGTC | CACTTGCATAGTTCTGCATTG |
| 30 | RM517* | 3 | GGCTTACTGGCTTCGATTTG | CGTCTCCTTTGGTTAGTGCC |
| 31 | RM251 | 3 | GAATGGCAATGGCGCTAG | ATGCGGTTCAAGATTCGATC |
| 32 | RM156* | 3 | GCCGCACCCTCACTCCCTCCTC | TCTTGCCGGAGCGCTTGAGGTG |
| 33 | RM16* | 3 | CGCTAGGGCAGCATCTAAA | AACACAGCAGGTACGCGC |
| 34 | RM55* | 3 | CCGTCGCCGTAGTAGAGAAG | TCCCGGTTATTTTAAGGCG |
| 35 | RM1352 | 3 | ACGAGTTGTACTCTGGTTGC | TCTCGGTTTTTATCTTGCTG |
| 36 | RM514 | 3 | AGATTGATCTCCCATTCCCC | CACGAGCATATTACTAGTGG |
| 37 | RM565 | 3 | AGTAACGAGCATAGCAGGCG | GCAAAGCCTTCAGGAATCAG |
| 38 | RM570 | 3 | GTTCTTCAACTCCCAGTGCG | TGACGATGTGGAAGAGCAAG |
| 39 | RM85* | 3 | CCAAAGATGAAACCTGGATTG | GCACAAGGTGAGCAGTCC |
| 40 | RM551* | 4 | AGCCCAGACTAGCATGATTG | GAAGGCGAGAAGGATCACAG |
| 41 | RM261* | 4 | CTACTTCTCCCCTTGTGTCG | TGTACCATCGCCAAATCTCC |
| 42 | RM32 | 4 | AGTCTACGTGGTGTACACGTGG | TGCGGCCTGCCGTTTGTGAG |
| 43 | RM307 | 4 | GTACTACCGACCTACCGTTCAC | CTGCTATGCATGAACTGCTC |
| 44 | RM6997 | 4 | CAACGCGGCAGTAAATTTGC | GGCCTTGTCAGTCTACATGC |
| 45 | RM273 | 4 | GAAGCCGTCGTGAAGTTACC | GTTTCCTACCTGATCGCGAC |
| 46 | RM252 | 4 | TTCGCTGACGTGATAGGTTG | ATGACTTGATCCCGAGAACG |
| 47 | RM3276 | 4 | TCCGTCTCGACTCTTCCATC | GATGAGACACCACGGACATG |
| 48 | RM348* | 4 | CCGCTACTAATAGCAGAGAG | GGAGCTTTGTTCTTGCGAAC |
| 49 | RM507 | 5 | CTTAAGCTCCAGCCGAAATG | CTCACCCTCATCATCGCC |
| 50 | RM122 | 5 | GAGTCGATGTAATGTCATCAGTGC | GAAGGAGGTATCGCTTTGTTGGAC |
| 51 | RM413 | 5 | GGCGATTCTTGGATGAAGAG | TCCCCACCAATCTTGTCTTC |
| 52 | RM574 | 5 | AAACTAGCCACGGTTTGGTAGGG | AGGGTGGCAGGGATGTAATTTCC |
| 53 | RM8039 | 5 | CGTACGTACTTATATCTCAT | AAATCTAATGTATCTGAGGT |
| 54 | RM5140 | 5 | GACGAGGTTGTTTATTAGTG | CTTATTTTCACGTGTACGTT |
| 55 | RM146 | 5 | CTATTATTCCCTAACCCCCATACCCTCC | AGAGCCACTGCCTGCAAGGCCC |
| 56 | RM163 | 5 | ATCCATGTGCGCCTTTATGAGGA | CGCTACCTCCTTCACTTACTAGT |
| 57 | RM178 | 5 | TCGCGTGAAAGATAAGCGGCGC | GATCACCGTTCCCTCCGCCTGC |
| 58 | RM31* | 5 | GATCACGATCCACTGGAGCT | AAGTCCATTACTCTCCTCCC |
| 59 | RM13 | 5 | TCCAACATGGCAAGAGAGAG | GGTGGCATTCGATTCCAG |
| 60 | RM586 | 6 | ACCTCGCGTTATTAGGTACCC | GAGATACGCCAACGAGATACC |
| 61 | RM3414 | 6 | TAGGGCAATTGTGCAAGTGG | TTGGGAATTGGGTAGGACAG |
| 62 | RM510 | 6 | AACCGGATTAGTTTCTCGCC | TGAGGACGACGAGCAGATTC |
| 63 | RM204* | 6 | GTGACTGACTTGGTCATAGGG | GCTAGCCATGCTCTCGTACC |
| 64 | RM454* | 6 | CTCAAGCTTAGCTGCTGCTG | GTGATCAGTGCACCATAGCG |
| 65 | RM162 | 6 | TTGTTCCAGTTCAGGTCTTGTGC | CCCTACAAACACCATAAGAAGCAACC |
| 66 | RM340 | 6 | GGTAAATGGACAATCCTATGGC | GACAAATATAAGGGCAGTGTGC |
| 67 | RM427 | 7 | TCACTAGCTCTGCCCTGACC | TGATGAGAGTTGGTTGCGAG |
| 68 | RM21078 | 7 | CAAGCTGCCGTGTTCTACTGG | GCACACAACAAGAGACAGTAACATGC |
| 69 | RM125* | 7 | ATCAGCAGCCATGGCAGCGACC | AGGGGATCATGTGCCGAAGGCC |
| 70 | RM5436 | 7 | CAAAGGGGGTGTCCTCTATG | GTTGCTCGTCCTACATGTGC |
| 71 | RM542 | 7 | TGAATCAAGCCCCTCACTAC | CTGCAACGAGTAAGGCAGAG |
| 72 | RM214* | 7 | CTGATGATAGAAACCTCTTCTC | AAGAACAGCTGACTTCACAA |
| 73 | RM11 | 7 | TCTCCTCTTCCCCCGATC | ATAGCGGGCGAGGCTTAG |
| 74 | RM455 | 7 | AACAACCCACCACCTGTCTC | AGAAGGAAAAGGGCTCGATC |
| 75 | RM1132 | 7 | ATCACCTGAGAAACATCCGG | CTCCTCCCACGTCAAGGTC |
| 76 | RM118 | 7 | CCAATCGGAGCCACCGGAGAGC | CACATCCTCCAGCGACGCCGAG |
| 77 | RM248* | 7 | TCCTTGTGAAATCTGGTCCC | GTAGCCTAGCATGGTGCATG |
| 78 | RM408 | 8 | CAACGAGCTAACTTCCGTCC | ACTGCTACTTGGGTAGCTGACC |
| 79 | RM152* | 8 | AAGGAGAAGTTCTTCGCCCAGTGC | GCCCATTAGTGACTGCTCCTAGTCG |
| 80 | RM137 | 8 | GACATCGCCACCAGCCCACCAC | CGGGTGGTCCCCGAGGATCTTG |
| 81 | RM3819 | 8 | ACCTCACCTGTGGATCTTGG | CAATCCCCTTCTCTCCTTCC |
| 82 | RM25* | 8 | GGAAAGAATGATCTTTTCATGG | CTACCATCAAAACCAATGTTC |
| 83 | RM1384 | 8 | TTAATCCATCCTGTAGCTGG | TCGCTATCAACACTACCTGC |
| 84 | RM223* | 8 | GAGTGAGCTTGGGCTGAAAC | GAAGGCAAGTCTTGGCACTG |
| 85 | RM284 | 8 | ATCTCTGATACTCCATCCATCC | CCTGTACGTTGATCCGAAGC |
| 86 | RM210* | 8 | TCACATTCGGTGGCATTG | CGAGGATGGTTGTTCACTTG |
| 87 | RM149 | 8 | GCTGACCAACGAACCTAGGCCG | GTTGGAAGCCTTTCCTCGTAACACG |
| 88 | RM433 | 8 | TGCGCTGAACTAAACACAGC | AGACAAACCTGGCCATTCAC |
| 89 | RM316 | 9 | CTAGTTGGGCATACGATGGC | ACGCTTATATGTTACGTCAAC |
| 90 | RM434* | 9 | GCCTCATCCCTCTAACCCTC | CAAGAAAGATCAGTGCGTGG |
| 91 | RM410 | 9 | GCTCAACGTTTCGTTCCTG | GAAGATGCGTAAAGTGAACGG |
| 92 | RM257* | 9 | CAGTTCCGAGCAAGAGTACTC | GGATCGGACGTGGCATATG |
| 93 | RM215* | 9 | CAAAATGGAGCAGCAAGAGC | TGAGCACCTCCTTCTCTGTAG |
| 94 | RM474 | 10 | AAGATGTACGGGTGGCATTC | TATGAGCTGGTGAGCAATGG |
| 95 | RM271 | 10 | TCAGATCTACAATTCCATCC | TCGGTGAGACCTAGAGAGCC |
| 96 | RM6100 | 10 | TCCTCTACCAGTACCGCACC | GCTGGATCACAGATCATTGC |
| 97 | RM484 | 10 | TCTCCCTCCTCACCATTGTC | TGCTGCCCTCTCTCTCTCTC |
| 98 | RM552 | 11 | CGCAGTTGTGGATTTCAGTG | TGCTCAACGTTTGACTGTCC |
| 99 | RM116 | 11 | TCACGCACAGCGTGCCGTTCTC | CAAGATCAAGCCATGAAAGGAGGG |
| 100 | RM536 | 11 | TCTCTCCTCTTGTTTGGCTC | ACACACCAACACGACCACAC |
| 101 | RM287* | 11 | TTCCCTGTTAAGAGAGAAATC | GTGTATTTGGTGAAAGCAAC |
| 102 | RM209* | 11 | ATATGAGTTGCTGTCGTGCG | CAACTTGCATCCTCCCCTCC |
| 103 | RM21* | 11 | ACAGTATTCCGTAGGCACGG | GCTCCATGAGGGTGGTAGAG |
| 104 | RM206 | 11 | CCCATGCGTTTAACTATTCT | CGTTCCATCGATCCGTATGG |
| 105 | RM224* | 11 | ATCGATCGATCTTCACGAGG | TGCTATAAAAGGCATTCGGG |
| 106 | RM144 | 11 | TGCCCTGGCGCAAATTTGATCC | GCTAGAGGAGATCAGATGGTAGTGCATG |
| 107 | RM19* | 12 | CAAAAACAGAGCAGATGAC | CTCAAGATGGACGCCAAGA |
| 108 | RM247 | 12 | TAGTGCCGATCGATGTAACG | CATATGGTTTTGACAAAGCG |
| 109 | RM277 | 12 | CGGTCAAATCATCACCTGAC | CAAGGCTTGCAAGGGAAG |
| 110 | RM519* | 12 | AGAGAGCCCCTAAATTTCCG | AGGTACGCTCACCTGTGGAC |
| 111 | RM235 | 12 | AGAAGCTAGGGCTAACGAAC | TCACCTGGTCAGCCTCTTTC |

*Polymorphic SSRs used in BC_2_F_2_ and BC_2_F_8_ generations for QTL mapping

S. No - Serial Number , Chr.- chromosome.
