# Supplementary material for: Identification of Major Effect QTLs for Agronomic Traits and CSSLs in Rice from Swarna/Oryza nivara Derived Backcross Inbred Lines
Source: Front Plant Sci. 2017 Jun 22;8:1027. doi: 10.3389/fpls.2017.01027 (PMC5480306; doi:10.3389/fpls.2017.01027)
Supplement: Supplementary file 3 [file Table_3.DOCX]

**Identification of major effect QTLs for agronomic traits and CSSLs in rice from Swarna/*Oryza nivara* derived backcross inbred lines**

**Supplementary Table.3** Correlation coefficients among nine yield related traits in two years

| **Trait** | **Season** | **DFF** | **DM** | **PH** | **NT** | **NPT** | **PW** | **YLDP** | **BM** | **BY** |
| --- | --- | --- | --- | --- | --- | --- | --- | --- | --- | --- |
| **DFF** | kh14 | 1 | **0.95**** | -0.12 | 0.16 | 0.13 | **-0.29**** | 0.01 | 0.08 | -0.18 |
|  | kh15 |  |  |  |  |  |  |  |  |  |
| **DM** | kh14 | **0.90**** | 1 | -0.14 | 0.12 | 0.08 | **-0.35**** | -0.08 | 0.04 | **-0.22*** |
|  | kh15 | **0.95**** |  |  |  |  |  |  |  |  |
| **PH** | kh14 | -0.04 | -0.05 | 1 | **-0.20*** | **-0.23*** | **0.40**** | **0.23*** | **0.45**** | 0.11 |
|  | kh15 | -0.12 | -0.12 |  |  |  |  |  |  |  |
| **NT** | kh14 | **0.17*** | 0.10 | -0.15 | 1 | **0.95**** | -0.12 | **0.43**** | **0.34**** | **0.28**** |
|  | kh15 | 0.13 | 0.13 | **-0.28**** |  |  |  |  |  |  |
| **NPT** | kh14 | 0.13 | 0.04 | **-0.20*** | **0.97**** | 1 | -0.12 | **0.38**** | **0.26**** | **0.32**** |
|  | kh15 | 0.16 | 0.16 | **-0.29**** | **0.91**** |  |  |  |  |  |
| **PW** | kh14 | -0.06 | -0.12 | **0.50**** | -0.16 | **-0.19*** | 1 | **0.42**** | 0.19 | 0.19 |
|  | kh15 | **-0.25**** | **-0.25**** | **0.26**** | -0.03 | -0.05 |  |  |  |  |
| **YLDP** | kh14 | 0.19* | 0.07 | **0.23*** | **0.59**** | **0.51**** | **0.31**** | 1 | **0.59**** | **0.29**** |
|  | kh15 | -0.16 | -0.16 | **0.19*** | 0.03 | 0.07 | **0.60**** |  |  |  |
| **BM** | kh14 | 0.14 | 0.10 | **0.38**** | **0.46**** | **0.35**** | 0.14 | **0.61**** | 1 | **0.25*** |
|  | kh15 | 0.09 | 0.09 | **0.61**** | 0.02 | 0.03 | **0.27**** | **0.54**** |  |  |
| **BY** | kh14 | 0.01 | -0.02 | -0.01 | **0.38**** | **0.41**** | -0.08 | **0.22*** | **0.22*** | 1 |
|  | kh15 | **-0.24**** | **-0.24**** | **0.30**** | 0.10 | 0.14 | **0.33**** | **0.54**** | **0.33**** |  |

Significance levels: **P* < 0.05, and ***P* < 0.01.Values in bold show correlation in both years. Wet season (Kh= kharif). Upper diagonal shows the correlation of mean data across years and lower diagonal present year wise correlations

DFF- days to 50% flowering, DM- days to maturity, PH- plant height, NT- number of tillers, NPT- number of productive tillers, PW- panicle weights, YLDP- yield per plant, BM- Biomass, BY- bulk yield.
