# Supplementary material for: Identification of Major Effect QTLs for Agronomic Traits and CSSLs in Rice from Swarna/Oryza nivara Derived Backcross Inbred Lines
Source: Front Plant Sci. 2017 Jun 22;8:1027. doi: 10.3389/fpls.2017.01027 (PMC5480306; doi:10.3389/fpls.2017.01027)
Supplement: Supplementary file 4 [file Table_4.DOCX]

**Identification of major effect QTLs for agronomic traits and CSSLs in rice from Swarna/*Oryza nivara* derived backcross inbred lines**

**Supplementary Table 4:** List of 22 CSSLs with significantly higher values over Swarna for five traits

| **S. No** | **CSSLs** | **Traits** |
| --- | --- | --- |
| 1 | 14_3S | PW |
| 2 | 192S | BM |
| 3 | 84S | YLDP, PW |
| 4 | 166-2-5S | PW |
| 5 | 166-23-1S | PW |
| 6 | 87-1S | BM |
| 7 | 252S | PW |
| 8 | 204S | BM |
| 9 | 40S | PW, DFF |
| 10 | 79S | PW, PH |
| 11 | 77S | BM, PH |
| 12 | 14S | DFF |
| 13 | 69S | PW, BM, |
| 14 | 10--2S | PW, YLDP, BM |
| 15 | 166-9S | YLDP |
| 16 | 112S | BM |
| 17 | 61S | BM |
| 18 | 220S | PW |
| 19 | 142S | BM |
| 20 | 148S | DFF, PH |
| 21 | 216S | BM |
| 22 | 247S | YLDP |

S. No - Serial Number, CSSLs - Chromosomal Segment Substitution Lines, DFF- Days to 50% flowering, PH- Plant height, PW- Panicle weight, YLDP- Yield per plant, BM- Biomass
