# Supplementary material for: Identification of Major Effect QTLs for Agronomic Traits and CSSLs in Rice from Swarna/Oryza nivara Derived Backcross Inbred Lines
Source: Front Plant Sci. 2017 Jun 22;8:1027. doi: 10.3389/fpls.2017.01027 (PMC5480306; doi:10.3389/fpls.2017.01027)

**Identification of major effect QTLs for agronomic traits and CSSLs in rice from Swarna/*Oryza nivara* derived backcross inbred lines**

**Supplementary Fig.2** Frequency distribution of nine yield and related traits in Swarna x *O. nivara* derived BILs in 2015. Arrow denotes Swarna value


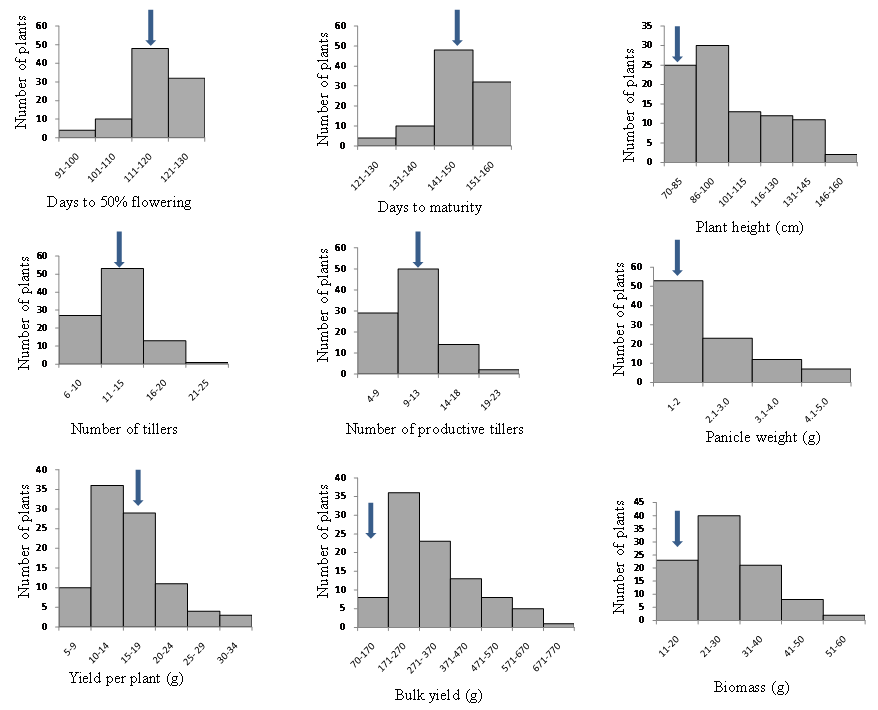

Supplement: Supplementary file 6 [file Table_6.DOCX]
